# Acute mountain sickness and sleep disturbances differentially influence cognition and mood during rapid ascent to 3000 and 4050 m

**DOI:** 10.14814/phy2.15175

**Published:** 2022-02-08

**Authors:** Peter S. Figueiredo, Ingrid V. Sils, Janet E. Staab, Charles S. Fulco, Stephen R. Muza, Beth A. Beidleman

**Affiliations:** ^1^ Biophysics and Biomedical Modeling Division U.S. Army Research Institute of Environmental Medicine Natick Massachusetts USA; ^2^ Thermal and Mountain Medicine Division U.S. Army Research Institute of Environmental Medicine Natick Massachusetts USA; ^3^ Military Performance Division U.S. Army Research Institute of Environmental Medicine Natick Massachusetts USA; ^4^ Strategic Science and Development Office U.S. Army Research Institute of Environmental Medicine Natick Massachusetts USA

**Keywords:** altitude, ANAM, hypobaric hypoxia, hypoxia, lowlanders

## Abstract

The impact of acute mountain sickness (AMS) and sleep disturbances on mood and cognition at two altitudes relevant to the working and tourist population is unknown. Twenty unacclimatized lowlanders were exposed to either 3000 m (*n* = 10; 526 mmHg) or 4050 m (*n* = 10; 460 mmHg) for 20 h in a hypobaric chamber. AMS prevalence and severity was assessed using the Environmental Symptoms Questionnaire (ESQ) and an AMS‐C score ≥ 0.7 indicated sickness. While sleeping for one night both at sea level (SL) and high altitude (HA), a wrist motion detector was used to measure awakenings (Awak, events/h) and sleep efficiency (Eff, %). If Eff was ≥85%, individuals were considered a good sleeper (Sleep+). Mood and cognition were assessed using the Automated Neuropsychological Assessment Metric and Mood Scale (ANAM‐MS). The ESQ and ANAM‐MS were administered in the morning both at SL and after 20 h at HA. AMS severity (mean ± SE; 1.82 ± 0.27 vs. 0.20 ± 0.27), AMS prevalence (90% vs. 10%), depression (0.63 ± 0.23 vs. 0.00 ± 0.24) Awak (15.6 ± 1.6 vs. 10.1 ± 1.6 events/h), and DeSHr (38.5 ± 6.3 vs. 13.3 ± 6.3 events/h) were greater (*p* < 0.05) and Eff was lower (69.9 ± 5.3% vs. 87.0 ± 5.3%) at 4050 m compared to 3000 m, respectively. AMS presence did not impact cognition but fatigue (2.17 ± 0.37 vs. 0.58 ± 0.39), anger (0.65 ± 0.25 vs. 0.02 ± 0.26), depression (0.63 ± 0.23 vs. 0.00 ± 0.24) and sleepiness (4.8 ± 0.4 vs. 2.7 ± 0.5) were greater (*p* < 0.05) in the AMS+ group. The Sleep− group, compared to the Sleep+ group, had lower (*p* < 0.05) working memory scores (50 ± 7 vs. 78 ± 9) assessed by the Sternberg 6‐letter memory task, and lower reaction time fatigue scores (157 ± 17 vs. 221 ± 22), assessed by the repeated reaction time test. Overall, AMS, depression, DeSHr, and Awak were increased (*p* < 0.05) at 4050 m compared to 3000 m. In addition, AMS presence impacted mood while poor sleep impacted cognition which may deteriorate teamwork and/or increase errors in judgement at HA.

## Graphical abstract

Acute mountain sickness (AMS) and awakenings were increased at 4050 m compared to 3000 m. In addition, AMS presence impacted mood while poor sleep impacted cognition which may deteriorate teamwork and/or increase errors in judgement at high altitude.

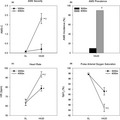

## 1 INTRODUCTION

Each year, millions of tourists (Basnyat, 2014), mountaineers (Keyes et al., 2016), athletes (Burtscher et al., 2018), military personnel (Caldwell et al., 2009), and workers (West, 2002) are rapidly exposed to hypobaric hypoxia equivalent to 3000–4000 m altitude. Decrements in mood (Bahrke & Shukitt‐Hale, 1993; Banderet et al., 2002), cognition (Banderet et al., 2002; McMorris et al., 2017; Virues‐Ortega et al., 2004), and sleep quality and quantity (Ainslie et al., 2013; Bloch et al., 2015) occur following rapid ascent to high altitude (HA) in unacclimatized lowlanders. In addition, acute mountain sickness (AMS), the most common altitude illness, increases in prevalence and severity following rapid ascent as a function of altitude attained (Beidleman et al., 2013). Symptoms, if severe, may completely incapacitate an individual's ability to perform their job (Roach et al., 2002). Symptoms of AMS, sleep quality and quantity, and impairments to mood and cognition are usually worst within the first 24–48 h of HA exposure and largely resolve thereafter (Banderet et al., 2002; Beidleman et al., 2013; Bloch et al., 2015; Virues‐Ortega et al., 2004). Furthermore, mood and cognition may be impacted by AMS and sleep disturbances independent of the impairments inflicted by hypobaric hypoxia alone (Aquino Lemos et al., 2012; Crowley et al., 1992; Heinrich et al., 2019). Although each of these topics has been reviewed individually in the literature (Ainslie et al., 2013; Bahrke & Shukitt‐Hale, 1993; Banderet et al., 2002; Bloch et al., 2015; Hackett & Roach, 2001; McMorris et al., 2017; Virues‐Ortega et al., 2004), a comprehensive examination of the influence of AMS and sleep disturbances on mood and cognition at two altitudes (e.g., 3000 and 4050 m) relevant to the tourist and working populations has not been conducted.

AMS is characterized by headache, gastrointestinal distress, lightheadedness, and fatigue (Hackett & Roach, 2001) and in some individuals poor sleep (Ainslie et al., 2013), and can be provoked by acute ascents to as low as 2500 m (Beidleman et al., 2013). Those with AMS typically demonstrate negative mood dispositions at HA (Crowley et al., 1992; Shukitt‐Hale et al., 1991) but the relationship between AMS and cognition is equivocal given that cognitive decrements typically occur prior to the manifestation of AMS and resolve while AMS is peaking (Bahrke & Shukitt‐Hale, 1993; Banderet et al., 2002). Disturbances in sleep can be observed as low as 1600 m (Latshang et al., 2013) and are nearly universal above 4000 m (Ainslie et al., 2013). Travelers to HA experience frequent arousals due to the characteristic waxing and waning of ventilation and often awake feeling unrefreshed (Ainslie et al., 2013; Bloch et al., 2015). Several studies at altitude (3650–4500 m) have demonstrated a negative impact of poor sleep on mood (Aquino Lemos et al., 2012; Heinrich et al., 2019) and cognition (Aquino Lemos et al., 2012; Kong et al., 2011).

Much of the previous research suggests a threshold altitude of 3000 m before decrements in mood and cognition emerge (Bahrke & Shukitt‐Hale, 1993; Banderet et al., 2002; Fowler et al., 1987; Li, Wu, Fu, Shen, Wu, et al., 2000; Li, Wu, Fu, Shen, Yang, et al., 2000), though many confounding factors contribute to discrepant findings between studies including altitude attained and the timing of measurements (Petrassi et al., 2012). Another cause of ambiguity in the mood and cognitive literature is the plethora of tests (≥100) utilized as well as the sheer number of mood and cognitive domains assessed (Petrassi et al., 2012; Virues‐Ortega et al., 2004). Cognitive domains range from simple to complex and are not uniformly affected by altitude exposure (Banderet et al., 2002; Petrassi et al., 2012). A strength of the Automated Neuropsychological Assessment Metric and Mood Scale (ANAM‐MS) is a decade's long record of use and direct linkage, for performance comparisons, to archival research literature (Lowe et al., 2007; Lowe & Reeves, 2002).

The altitude range from 3000 to 4000 m appears to be particularly relevant to the tourist and working populations mentioned above and encompasses the threshold where AMS, mood disturbances, cognitive impairments, and sleep disturbances are first observed (Ainslie et al., 2013; Banderet et al., 2002; Hackett & Roach, 2001). The purpose of this study was to examine the influence of AMS and sleep on mood and cognition at two altitudes (3000 and 4050 m) using the ANAM‐MS under controlled environmental, activity, and ascent conditions. We hypothesized that (1) altitude would impact AMS symptomatology and sleep disturbances with greater symptoms and disturbances at 4050 m compared to 3000 m, (2) altitude would impact mood and cognition with greater disturbances at 4050 m compared to 3000 m, (3) those individuals susceptible to AMS, regardless of altitude, would experience greater decrements in mood and cognition at HA, and (4) those individuals susceptible to sleep disturbances, regardless of altitude, would experience greater decrements in mood and cognition at HA.

## 2 METHODS AND MATERIALS

### 2.1 Volunteers

This study was approved by the Institutional Review Board at the U.S. Army Research Institute of Environmental Medicine (USARIEM) and conformed to the Declaration of Helsinki. All volunteers provided written and verbal acknowledgment of their informed consent and were made aware of their right to withdraw without prejudice at any time. Investigators adhered to the policies for protection of human subjects as prescribed in Department of Defense Instruction 3216.02, and the research was conducted in adherence with 32 CFR Part 219. All volunteers were healthy, well nourished, physically active, non‐smokers with hematologic and ferritin values in the normal range. Age range was limited to 18–39 year olds, and all exhibited normal pulmonary function at sea level (SL, 756 mmHg). None had been diagnosed with a sleep disorder. All volunteers were born at <1500 m, were currently living at SL, and had no recent exposures to HA. Data was lost for a volunteer in the 3000 m group due to a technical error and this participant was dropped from analysis. Physical characteristics were determined at SL and are presented in Table 1 for the entire cohort as well as by altitude group.

**TABLE 1:** Demographic sea‐level characteristics of both groups of unacclimatized lowlanders (mean ± SE)

	Sex (M/F)	Age (year)	Height (cm)	Weight (kg)	VO_2peak_ (ml kg^−1^ min^−1^)
3000 m (*n* = 10)	9/1	21.0 ± 0.8	173.5 ± 2.5	81.4 ± 4.2	46.1 ± 2.0
4050 m (*n* = 10)	8/2	23.6 ± 1.9	173.4 ± 3.4	72.6 ± 3.9	45.5 ± 1.5
Combined (*n* = 20)	17/3	22.3 ± 1.0	173.45 ± 2.1	77.0 ± 3.0	45.8 ± 1.2

### 2.2 Study design

The study was a randomized, single‐blind study conducted over a 14‐day period in two phases in the following order: (1) 4 days at SL (50 m, 756 mmHg) for baseline testing and (2) a 20 h over‐night, exposure to 3000 m (526 mmHg) or 4050 m (460 mmHg) altitude in a hypobaric chamber. AMS, mood states, and cognitive performance were assessed every morning over 4 days at SL to achieve a stable baseline. The mean of the third and fourth administration at SL was used as the baseline record. The following week, volunteers rapidly ascended to their assigned altitude in a hypobaric chamber over a 15‐min period (regardless of altitude) at around noon and left the hypobaric chamber ~20 h later. Following the initial ascent and a rest period, all volunteers underwent 3 h of walking exercise (40 min on and 20 min off) on a treadmill conducted at ~40% of SL peak oxygen uptake (VO_2peak_) to simulate light on/off exercise under controlled environmental (20 ± 2°C; 40 ± 5%) conditions. Mood, cognitive performance, and AMS assessments were obtained the following morning after sleeping overnight in the hypobaric chamber, 20 h after ascent (HA20). All measurements were taken at the same time, regardless of group, and all volunteers were awakened at ~6:30 am to begin measurements. None of the volunteers used any type of medication. During both phases of the study, volunteers had ad libitum access to the foods and liquids available. Stouffers frozen dinners, or Hot Pockets supplemented with snack bars, fresh fruits, juices, milk and sport beverages were available.

### 2.3 Study measures

#### 2.3.1 Peak oxygen uptake

An incremental, progressive exercise bout to volitional exhaustion on a treadmill was used to assess VO_2peak_ during the SL baseline phase. Measurements of O_2_ uptake were obtained using a metabolic cart (True Max 2400; Parvo Medics) using a previously described protocol (Kenefick et al., 2019). The data obtained fromVO_2peak_ testing was used to calculate the treadmill speeds and grades to elicit the desired 40% SL VO_2peak_ during the walking exercise.

### 2.4 Acute mountain sickness

The prevalence and severity of AMS was determined from information gathered using the Environmental Symptoms Questionnaire (ESQ; Sampson et al., 1983). The shortened electronic version of the ESQ, which is a self‐reported 11‐question inventory, was used to quantify a weighted AMS cerebral factor score (AMS‐C), and AMS was judged to be present if AMS‐C was ≥0.7 (Beidleman et al., 2007). The ESQ was utilized instead of the Lake Louise Questionnaire (LLQ) due to the discord in AMS diagnosis criteria from LLQ scoring. Some researchers use a cutoff value of ≥3 plus headache to define AMS (Roach et al., 1993) while others utilize ≥4 plus headache (Maggiorini et al., 1998) or ≥5 plus headache (Wagner et al., 2012). Directly after completion of the questionnaire, resting heart rate (HR) and pulse arterial oxygen saturation (SpO_2_) were assessed with pulse oximetry (Model 8600; Nonin Medical) for 2 min and the mean for each was calculated.

### 2.5 Sleep assessment

An actigraph (Motion Logger, Ambulatory Monitoring, Inc.) was used to differentiate sleep from wakefulness based on wrist movement. Estimation of sleep by actigraphy has a high level of correlation with sleep estimation by polysomnography (Cole et al., 1992; Souza et al., 2003). Volunteers wore the actigraph on the wrist of their dominant hand and pressed an event button when they lay down to sleep and again on waking up in the morning. Recordings between the two events were analyzed for sleep awakenings (Awak, events/h), total time in bed (min), total sleep time (min), and sleep efficiency (Eff, %). Eff was calculated as total sleep time divided by the total time in bed and multiplied by 100. Data were recorded using zero crossing mode (ZCM) in 1‐min epochs. Automated analysis was performed using the Cole‐Kripke algorithm on the ZCM channel at a 1‐min sample rate (Action 4 version 1.13; Ambulatory Monitoring Inc.; Cole et al., 1992; Souza et al., 2003). If the Eff was ≥85%, individuals were considered a good sleeper (Sleep+) and otherwise considered a poor sleeper (Sleep−; Ohayon et al., 2017).

A pulse oximeter, worn on the non‐dominant hand, was used to measure nocturnal SpO_2_ and HR (Nonin 3100 WristOx; Nonin Medical, Inc.). Study volunteers were instructed to put on the pulse oximeter when they went to bed and to remove the pulse oximeter the next morning when they got out of bed. Compatible software was used to determine mean sleep pulse oxygen saturation (S‐SpO_2_, %), sleep heart rate (S‐HR, beats/min), and relative desaturations (DeSHr, events/h) defined as >4% drop in oxygen saturation for a minimum of 10 s (nVISION Version 6.3j, Nonin Medical, Inc.; Ruehland et al., 2009). All volunteers wore the same actigraph and pulse oximeter throughout the study.

### 2.6 Mood state

Mood states were assessed using the Automated Neuropsychological Assessment Metrics Mood Scale (ANAM‐MS), a valid and reliable assessment tool when compared to concurrent, well‐validated measures of mood (Johnson et al., 2008). The ANAM‐MS was given in computerized format using a 0–6, visual analog Likert scale to express how well 42 adjectives describe the volunteers' mood state. These 42 adjectives represent seven dimensions of mood including vigor, restlessness, depression, anger, fatigue, anxiety, and happiness. Mean ratings are computed for each scale with higher values reflecting a greater degree of endorsement of each of the mood states. The Stanford sleepiness scale is validated (Hoddes et al., 1973) and was incorporated in the ANAM‐MS. The Stanford Sleepiness Scale uses a 1–7, visual analog Likert scale to quantify volunteers' sleepiness.

### 2.7 Cognitive performance assessment

Ten ANAM modules (Reeves et al., 2002) were utilized to assess cognitive performance: code substitution learning (CDS), code substitution delayed memory (CDD), match to sample, mathematical processing, go‐no‐go, simple reaction time, procedural reaction time, simultaneous spatial processing, Sternberg 6‐letter memory (ST6), and simple reaction time 2 (SRT2). Throughput scores, which take into consideration both speed and accuracy, or percent correct (for tests without throughput scores) were used for the analyses. Time to complete all cognitive tests differed for each individual but was approximately 30 min from start to finish. These tests and the cognitive domains assessed have been described previously (Reeves et al., 2007) (Table 2).

**TABLE 2:** Automated Neuropsychological Assessment Metric (ANAM) module clarifications

Module	Cognitive domain
CDS	Visual search, sustained attention, working memory
CDD	Sustained attention, working memory, learning
M2S	Spatial processing, visuospatial working memory
MTH	Computational skills, concentration, working memory
GNG	Executive function, decision‐making, processing speed
SRT	Processing speed
PRT	Processing speed, attention
SPD	Spatial processing
ST6	Working Memory
SRT2	Cognitive fatigue, processing speed

### 2.8 Statistical analysis

Data normality was tested using the Shapiro Wilk test and if parameters failed to meet normality, data was log transformed for analysis. Homogeneity of variance was checked using Levene's test and none of the variables violated this assumption after log transformation. A repeated‐measures mixed ANOVA (SPSSv24; IBM) with one independent factor (group; 3000 and 4050 m) and one repeated‐measures factor (condition; SL and HA20) was used to analyze the AMS‐C, sleep, mood and cognition data. In addition, repeated‐measures mixed ANOVAs were used to analyze the data with AMS group (AMS+ and AMS−) and sleep group (Sleep+ and Sleep−), as independent factors, regardless of altitude. Tukey post‐hoc tests were used to follow up on main and interaction effects. AMS prevalence was analyzed using SAS PROC GLIMMIX (SAS 9.2, Cary, NC) across conditions (SL and HA20). Pearson correlation coefficients were utilized to examine relationships between AMS‐C and mood/cognitive performance scores; and, between sleep measures and mood/cognitive performance scores. Sample‐size analysis indicated that a sample size of 8–10 individuals would detect a 0.7 point difference in AMS‐C, 50 ms difference in simple reaction time, and 100 point difference in code substitution throughput with 80% power at the 0.05 confidence level (Heinrich et al., 2019; Roach et al., 2014; Seo et al., 2015). Statistical significance was set at *p* < 0.05. All data are reported as mean ± SE.

## 3 RESULTS

### 3.1 Impact of altitude on AMS and resting physiologic measures

**FIGURE 1:**
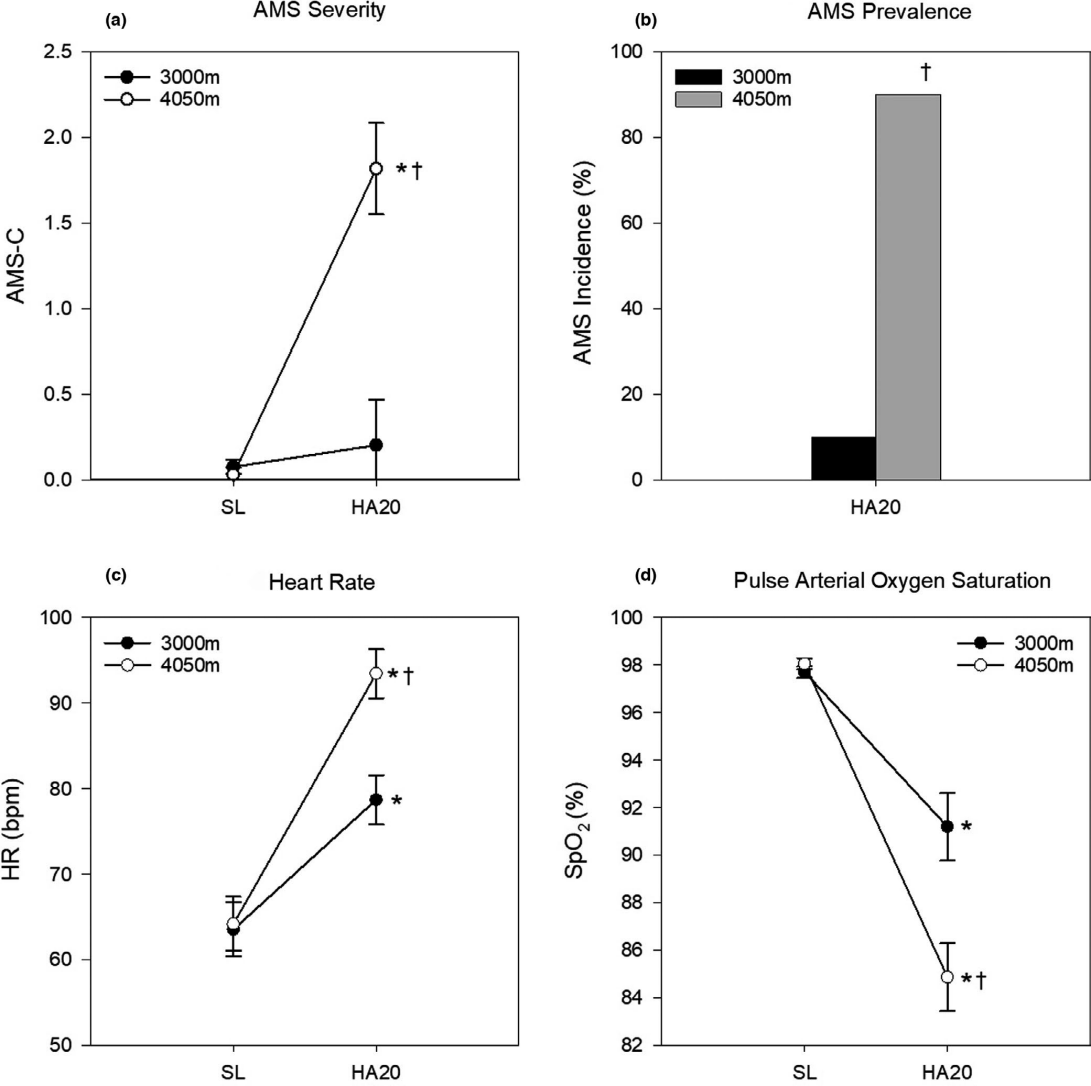
Impact of altitude on acute mountain sickness (AMS) and resting physiologic measures. All data presented as mean ± SE. (a) AMS severity (AMS‐C), (b) AMS prevalence (%), (c) resting heart rate (bpm), (d) resting SpO2 (%) at sea level (SL) and after 20 h of simulated altitude exposure (HA20). *p < 0.05 from SL; ^†^ *p* < 0.05 between groups at HA20

Figure 1 presents the prevalence and severity of AMS, resting HR, and resting SpO_2_ for both altitude groups at SL and HA20. The AMS prevalence and severity was higher (*p* < 0.0002) in the 4050 m compared to 3000 m group. Both groups demonstrated significant increases (*p* < 0.05) in resting HR and decreases in SpO_2_. In addition, resting HR was 19% higher (*p* = 0.009) and SpO_2_ was 6% lower (*p* = 0.001) in the 4050 m compared to the 3000 m group.

### 3.2 Impact of altitude on cognitive performance and mood states

Table A1 presents the ANAM‐MS results for cognition and mood for both altitude groups (3000 m vs. 4050 m) in each condition (SL and HA20). None of the cognitive performance modules expressed a significant group, condition, or interaction effect. The 4050 m group exhibited higher (*p* = 0.005) ratings of depression than the 3000 m group at HA20. Ratings of fatigue, restlessness, sleepiness increased while vigor decreased (*p* < 0.05) in the 4050 m group from SL to HA20.

### 3.3 Impact of altitude on sleep disturbances

Table A1 presents the sleep data for both altitude groups in each condition (SL and HA20). Sleep was negatively impacted (*p* < 0.05) at HA20 in both groups but more so in the 4050 m group. The S‐SpO_2_ was 15% lower (*p* = 0.0002) and S‐HR was 28% higher (*p* = 0.0002) in the 4050 m group compared to the 3000 m group at HA20. Awak (events/h) were 54% higher (*p* = 0.03), DeSHr (events/h) were 300% higher (*p* = 0.004), and sleep Eff (%) was 17% lower (*p* = 0.03) in the 4050 m group compared to the 3000 m group at HA20.

### 3.4 Impact of AMS presence on cognitive performance and mood states

One participant from the 3000 m group and nine participants from the 4050 m group were a posteriori allocated to the AMS+group while eight participants from the 3000 m group and one participant from the 4050 m group were allocated to the AMS− group. Table A2 presents the ANAM‐MS results for cognition and mood by condition (SL and HA20) using AMS presence (AMS+ and AMS−) as the group factor. None of the cognitive performance modules expressed a significant group, condition, or interaction effect. Anger, depression, fatigue and sleepiness were higher (*p* < 0.05) in the AMS+compared to the AMS‐ group. Restlessness increased while vigor decreased (*p* < 0.05) from SL to HA20 only in the AMS+group. Among all study volunteers, AMS‐C significantly correlated with anxiety (*r* = 0.60, *p* = 0.007), depression (*r* = 0.59, *p* = 0.008), restlessness (*r* = 0.63, *p* = 0.004), vigor (*r* = −0.47, *p* = 0.04), and sleepiness (*r* = 0.70, *p* = 0.001) at HA20.

### 3.5 Impact of sleep on cognitive performance and mood states

Six participants from the 3000 m and one from the 4050 m group were a posteriori allocated to the Sleep+ group while three participants from the 3000 m group and nine participants from the 4050 m group were allocated to the Sleep− group. Table A3 presents the ANAM‐MS results for cognition and mood by condition (SL and HA20) using sleep quality (Sleep+ and Sleep−) as the group factor. The ST6 working memory scores were 56% higher (*p* = 0.020) and SRT2 scores were 29% higher (*p* = 0.048) in the Sleep+ compared to the Sleep− group at HA20. Sleepiness and restlessness was increased, and vigor was decreased (*p* < 0.05) from SL to HA20 only in the Sleep− group. Sleepiness was higher and vigor was lower (*p* < 0.05) in the Sleep− group compared to the Sleep+ group at HA20. Among all study volunteers, Awak was significantly correlated with learning, as measured by CDD (*r* = −0.51, *p* = 0.025).

## 4 DISCUSSION

These two altitudes were selected as they were expected to impose a moderate and large hypoxic strain and induce differences in AMS prevalence and severity (Beidleman et al., 2013), mood and cognition (Li, Wu, Fu, Shen, Wu, et al., 2000; Li, Wu, Fu, Shen, Yang, et al., 2000; Shukitt‐Hale et al., 1998), and sleep disturbances (Ainslie et al., 2013; Bloch et al., 2015). The desired effect was attained with a clear divergence between altitude groups in the prevalence and severity of AMS, resting HR and SpO_2_, S‐HR and S‐SpO_2_, Awak, Eff, and DeSHR at HA20. Our findings support some but not all of our hypotheses. Altitude impacted AMS symptomatology and sleep disturbances with greater symptoms and disturbances at 4050 m compared to 3000 m but had no impact on mood and cognition, except for an increase in depression in the 4050 m group. Those individuals susceptible to AMS, regardless of altitude, did not experience greater decrements in cognition but fatigue, anger, depression, and sleepiness were increased in the AMS+compared to AMS‐ individuals. Last, those individuals susceptible to sleep disturbances, regardless of altitude, demonstrated poorer working memory performance and decreased reaction time as well as increased ratings of sleepiness and decreased ratings of vigor at HA20. Increased AMS severity was associated with mood disturbances in five out of the eight mood indices while increases in Awak were associated with impairments in working memory/learning (CDD) in participants, regardless of grouping.

### 4.1 Altitude: Impact on AMS, sleep, cognition and mood

The foremost consideration when traveling or working at altitude is the altitude attained given the growing reduction in O_2_ delivery to the working muscles and brain with increasing altitude (Hackett & Roach, 2001). Our measures of resting HR and SpO_2_ (Crowley et al., 1992; Kenefick et al., 2019), AMS prevalence and severity (Beidleman et al., 2013; Kenefick et al., 2019), and all sleep variables are in agreement with previously published reports (Aquino Lemos et al., 2012; Heinrich et al., 2019; Latshang et al., 2013) at similar altitudes. While it has been demonstrated that men suffer from poorer sleep at altitude than women (Ainslie et al., 2013), our female volunteers were equally spread throughout the groups having minimal impact on analysis. It is worth noting that the simulated activity at altitude was standardized to ⁓40% of SLVO_2peak_and that the 4050 m was therefore working at a higher relative exercise intensity in the early hours of altitude exposure. This confounding influence may (Roach et al., 2000) or may not (Schommer et al., 2012) have contributed to higher AMS levels in the 4050 m group compared to the 3000 m group.

There was no effect of altitude on cognitive performance in the current study despite the wide array of cognitive domains tested with the ANAM‐MS. A priori, we hypothesized that various ANAM modules would express a greater decrement in the 4050 m group as it has been generally accepted that cognitive performance declines above 3000 m (Banderet et al., 2002; Cudaback, 1984; Fowler et al., 1987; Li, Wu, Fu, Shen, Yang, et al., 2000). Complex and unfamiliar tasks have been shown to be the most susceptible to hypoxia compared to simple or previously mastered tasks (Banderet et al., 2002; Cudaback, 1984). Lack of agreement among studies may be related to ascent profiles, duration of exposure, cognitive tests used, medications and uncontrolled environmental conditions (Petrassi et al., 2012).

Previous research provides strong evidence for the direct role of hypoxemia, expressed as SpO_2_ (Fowler et al., 1987; Li, Wu, Fu, Shen, Yang, et al., 2000; McMorris et al., 2017; Van der Post et al., 2002) on cognitive performance decrements at altitude. The apparent relationship between SpO_2_ and cognitive performance (Banderet et al., 2002; Fowler et al., 1987; Li, Wu, Fu, Shen, Yang, et al., 2000; Van der Post et al., 2002) was not prominent within the current study with no correlation between SpO_2_ and ANAM results at HA20. Previous researchers have found a threshold for cognitive performance decrements in acute hypoxia (≤120 min) at SpO_2_ levels below 82% (Fowler et al., 1987; Li, Wu, Fu, Shen, Yang, et al., 2000; Van der Post et al., 2002). By HA20, the 3000 and 4050 m groups had SpO_2_ levels of 91 and 85%, respectively, most likely due to ventilatory acclimatization. This may explain why our results deviated from previous literature and our hypothesis. Our volunteers may have acclimated over the 20 h of altitude exposure and may have compensated enough that cognitive performance decrements were marginal at the time of measurement.

Our findings of mood disturbances during HA exposure are in agreement with the majority of literature on the subject (Shukitt & Banderet, 1988; Shukitt‐Hale et al., 1990, 1998). Five out of the eight mood indices were negatively disturbed at HA20 in the 4050 m group. In concert with our observations, other researchers have also suggested the threshold for mood disturbances to be >3000 m (Banderet et al., 2002; Li, Wu, Fu, Shen, Wu, et al., 2000; Shukitt‐Hale et al., 1990). Changes in sleepiness and vigor in the 4050 m group supported our hypothesis while no changes in mood in the 3000 m group was contrary to our hypothesis.

A possible factor for the lack of change in mood in the 3000 m group is the timing of measurements and lack of AMS. Li, Wu, Fu, Shen, Wu, et al. (2000) found that mood states deviated from baseline at 2800 m but returned to baseline over a 1 h exposure while all mood disturbances at 3600–4400 m lasted throughout a 1 h exposure. In the current study, the volunteers acclimatized for 20 h which allowed for the confounding impact of acclimatization. In addition, Crowley et al. (1992) found that volunteers afflicted by AMS accounted for most of the overall changes in mood over a 2.5 day exposure to 4300 m.

### 4.2 AMS susceptibility: Impact on cognition and mood

At HA20, our volunteers were equally split as “sick” and “not sick” with the afflicted group demonstrating greater mood disturbances. When AMS group was utilized as the independent grouping factor, six out of eight of the moods demonstrated significant differences. When all volunteers were pooled, the moderate correlations between mood and AMS‐C score supported the expected relationship. Other researchers have reported similar relationships between AMS and mood (Crowley et al., 1992; Forster, 1985; Shukitt‐Hale et al., 1990, 1991). This relationship is supported by the similar time lines of AMS and mood disturbances, which both tend to be fully ameliorated after ⁓48–72 h of exposure (Shukitt & Banderet, 1988).

AMS is associated with an ascent too fast for the body to acclimatize to the rapid drop in PaO_2_ (Berger et al., 2019; Virues‐Ortega et al., 2004). Neuropsychological alterations are associated with a reduced PaO_2_ in the organism independent of acclimatization (McMorris et al., 2017; Virues‐Ortega et al., 2004). Hypobaric hypoxia can cause both AMS and cognitive performance decrements, however they can occur independently of each other with large individual variability (Berger et al., 2019). As the differing timelines suggest (Banderet et al., 2002), AMS development and cognitive performance decrements do not seem to be related in the current study. While some have found correlations between AMS and cognitive performance decrements (Forster, 1985; Shukitt‐Hale et al., 1991), others have not (Abraini et al., 1998; Beidleman et al., 2017; Kramer et al., 1993) and it is generally believed they are not directly related (Shukitt‐Hale et al., 1991; Virues‐Ortega et al., 2004).

### 4.3 Sleep: Impact on cognitionand mood

At HA, the groups were split into good and poor sleepers as measured using an established sleep efficiency cutoff value (Ohayon et al., 2017). The impact of sleep groups on next day sleepiness was anticipated. The ANAM modules that reflect working memory (ST6) and mental fatigue (SRT2) were impacted by poor sleep. These findings are similar to what has been observed in other studies at both SL (Findley et al., 1986) and HA (Aquino Lemos et al., 2012; Kong et al., 2011). When comparing sleep apnea patients with and without accompanying hypoxemia, Findley et al. (1986) found that the sleep apnea with hypoxemia group had significantly lower scores in four of eight cognitive performance tests and had double the desaturations per hour during sleep. Additionally, in a cohort of 230 soldiers stationed at HAs (3500–5800 m), scores on the Pittsburg Sleep Quality Index, inversely correlated with intelligence quotient (IQ) score and both long‐ and short‐term memory (Kong et al., 2011). In this study, poor sleep quality was an independent predictor of impaired IQ and digit symbol score (short‐term memory) (Kong et al., 2011). Roach et al. also found a decrement in SRT2, a measure of cognitive fatigue, at 5200 m with no decrements reported in SRT which is consistent with our results (Roach et al., 2014).

Similarly, the Sleep+ and Sleep− groups in the current study differed in DeSHr at HA20 (9.7 events/h vs. 33.4 events/h) while both groups experienced similar levels of nocturnal hypoxemia (83% vs. 79%,). Like sleep apnea at SL, the periodic breathing associated with sleep at altitude causes intermittent hypoxia (IH) beyond the continuous hypoxia experienced from altitude alone (Bloch et al., 2015). IH has been shown to have beneficial adaptations and maladaptations depending on the frequency, severity, and duration of the desaturations (Almendros et al., 2014). While continuous hypoxia promotes increased expression and activity in both hypoxia‐inducible factor 1‐alpha (HIF‐1α) and HIF‐2α, acute IH upregulates HIF‐1α and downregulates HIF‐2α (Nanduri et al., 2009). Within the current study, we can speculate that the stronger overnight stimulus of IH in the Sleep− group, characterized by increased DeSHr, may contribute to increases in ROS via down‐regulation of HIF‐2α and therefore insufficient transcription of antioxidative enzymes (Almendros et al., 2014). Increased ROS promotes a proinflammatory response, neuronal apoptosis and microglial activation which may contribute to cognitive deficits (Almendros et al., 2014) and possibly the significantly lower measures of working memory and reaction time in the Sleep− group.

The suggested relationship between impaired sleep and cognitive performance at altitude has inconsistent support from the literature. While studying the efficacy of adaptive servoventilation versus supplemental oxygen maintaining SpO_2_ > 95%, one study observed impaired sleep, cognitive performance, and disturbed mood at 3800 m (Heinrich et al., 2019). Supplemental oxygen was most effective at improving sleep quality and eliminating desaturations. Both interventions improved mood, though neither intervention had an impact on cognitive impairment which improved over subsequent days at altitude (Heinrich et al., 2019). On the other hand, in normobaric hypoxia equivalent to 4500 m, de Aquino Lemos et al. (2012) observed declines in both sleep and cognitive performance variables with correlations between them. Among these correlations, apnea/hypopnea index, which integrates respiratory and desaturation events, correlated with working memory and inhibitory control (Aquino Lemos et al., 2012) supporting the detrimental impact of IH while sleeping during acute exposure to altitude.

### 4.4 Implications

Implications from this study apply to the millions of people that visit high terrestrial terrain (Basnyat, 2014; Burtscher et al., 2018; Keyes et al., 2016; West, 2002) or are exposed to hypobaric hypoxia (Caldwell et al., 2009; States & Regulations, 2018), but especially those individuals that have to work at HA such as military personnel, pilots, mine/telescope workers, and emergency search and rescue personnel. Small changes in cognition may have drastic effects in emergency situations and mood changes can impact team cohesiveness and willingness to complete the job. Military pilots can fly at cabin pressures up to 3000 m (States & Regulations, 2018) and ultra‐long haul flights (>12 h) are being employed more frequently to decrease the logistical burden of troop movement (Caldwell et al., 2009). AMS can occur within that short time frame and exacerbate the mood and cognitive changes induced by exposure to hypobaric hypoxia alone (Crowley et al., 1992; Forster, 1985; Shukitt‐Hale et al., 1991; Virues‐Ortega et al., 2004). Moreover, the quality of sleep that pilots experience during their assigned in‐flight sleep period is known to be poorer and fragmented compared to SL sleep (Signal et al., 2013). From these results, it appears that AMS symptoms have a greater negative impact on mood while poor sleep has a greater negative impact on cognition and both can be detrimental in a work situation.

### 4.5 Limitations

One limitation to the current study is the inability to discuss the time course of AMS, physiologic responses, and mood and cognition disturbances. With just two sampling periods, we cannot determine the effect acclimatization had on our measures. It would have added value to have some short‐term measures to see how mood and cognitive performance changed prior to AMS development and sleep; then compare those results to the HA20 time point. Having periodic blood samples to assess hematologic, metabolic gene expression, and ventilatory acclimatization would also improve the study and allow for less speculative discussion regarding the mechanism behind altered cognition after HA sleep.

## 5 CONCLUSION

The degree of altitude impacted both AMS and sleep with greater symptoms and disturbances, respectively, in the 4050 m group. AMS presence did not impact cognition, but anger, depression, fatigue, and sleepiness were greater in those with AMS while poor sleepers demonstrated a decrease in their working memory and reaction time scores. Overall, AMS presence impacted mood while poor sleep impacted cognition which may deteriorate teamwork and/or increase errors in judgement at HA.

## DISCLOSURES

No conflict of interest for any author.

## AUTHOR CONTRIBUTIONS

Data analysis, data interpretation, manuscript preparation, final manuscript approval: Peter S. Figueiredo. Data collection, final manuscript approval: Ingrid V. Sils. Data collection, final manuscript approval: Janet E. Staab. Study design, data collection, data interpretation, final manuscript approval: Charles S. Fulco. Study design, data collection, data interpretation, final manuscript approval: Stephen R. Muza. Study design, data collection, data analysis, manuscript preparation, final manuscript approval: Beth A. Beidleman.

## APPENDIX 1

**TABLE A1:** Condition = Sea level versus high altitude. Group = 3000 m versus 4050 m. Throughput or percent correct scores of ten Automated Neuropsychological Assessment Metrics (ANAM) modules, subjective ratings of eight mood states (ANAM‐MS), and sleep analysis variables

Group	3000 m (*n* = 9)		4050 m (*n* = 10)		Condition effects		Group effects		Condition × Group	
Condition	SL	HA20	SL	HA20	*F*	*p*	*F*	*p*	*F*	*p*
*ANAM modules*										
CDS	56 ± 4	51 ± 5	58 ± 3	53 ± 5	4.05	0.06	0.10	0.75	0.00	0.95
CDD	52 ± 5	51 ± 6	50 ± 5	50 ± 5	0.01	0.91	0.04	0.84	0.01	0.92
M2S	43 ± 6	37 ± 6	28 ± 6	31 ± 6	1.56	0.23	0.53	0.48	3.21	0.09
MTH	23 ± 3	22 ± 3	25 ± 3	23 ± 3	1.88	0.19	0.16	0.69	0.75	0.40
GNG	89 ± 2	85 ± 4	89 ± 2	86 ± 3	3.63	0.07	0.02	0.88	0.17	0.69
SRT	213 ± 11	210 ± 20	208 ± 11	207 ± 19	0.03	0.86	0.04	0.85	0.01	0.92
PRT	101 ± 5	94 ± 8	94 ± 5	92 ± 8	2.05	0.17	0.28	0.60	0.62	0.44
SPD	37 ± 3	37 ± 5	30 ± 2	37 ± 4	2.49	0.13	0.47	0.50	2.22	0.16
ST6	66 ± 5	58 ± 9	62 ± 4	62 ± 8	0.68	0.42	0.00	0.98	0.62	0.44
SRT2	209 ± 13	173 ± 22	187 ± 12	187 ± 21	1.26	0.28	0.09	0.76	2.07	0.17
*Mood scale*										
Anger	0.08 ± 0.04	0.07 ± 0.27	0.10 ± 0.04	0.60 ± 0.26	0.08	0.78	1.33	0.26	0.66	0.43
Anxiety	0.09 ± 0.06	0.11 ± 0.18	0.09 ± 0.06	0.40 ± 0.17	1.29	0.27	1.25	0.28	0.47	0.50
Depression	0.03 ± 0.03	0.00 ± 0.24	0.08 ± 0.03	0.63 ± 0.23* ^,^ ^†^	1.86	0.19	7.28	0.01	2.74	0.12
Fatigue	0.55 ± 0.16	1.00 ± 0.46	0.46 ± 0.15	1.79 ± 0.44*	11.78	0.00	0.77	0.39	2.84	0.11
Happiness	1.98 ± 0.59	1.38 ± 0.57	1.83 ± 0.56	1.15 ± 0.54	1.31	0.27	0.11	0.74	0.00	0.95
Restlessness	0.08 ± 0.05	0.22 ± 0.28	0.08 ± 0.05	0.78 ± 0.27*	6.90	0.02	1.83	0.19	2.18	0.16
Vigor	1.46 ± 0.45	1.09 ± 0.30	2.02 ± 0.43	0.69 ± 0.29*	11.39	0.00	0.02	0.88	3.63	0.07
Sleepiness	2.6 ± 0.3	3.2 ± 0.6	2.3 ± 0.2	4.3 ± 0.5*	9.04	0.01	0.74	0.40	3.84	0.07
*Sleep*										
S‐SpO_2_ (%)	97 ± 0.3	86 ± 1.3*	97 ± 0.3	75 ± 1.3* ^,^ ^†^	334.78	0.00	35.44	0.00	36.76	0.00
S‐HR (beats/min)	51 ± 2.0	65 ± 3.0*	58 ± 2.0 ^†^	83 ± 3.0* ^,^ ^†^	70.57	0.00	21.74	0.00	5.41	0.03
DeSHr (events/h)	0.1 ± 0.1	13.3 ± 6.3	0.2 ± 0.1	38.5 ± 6.3* ^,^ ^†^	32.91	0.00	8.07	0.01	7.82	0.01
Awak (events/h)	6.0 ± 0.9	10.1 ± 1.6*	4.6 ± 0.9	15.6 ± 1.6* ^,^ ^†^	55.93	0.00	1.79	0.20	11.68	0.00
Eff (%)	96.0 ± 1.4	87.0 ± 5.3	95.8 ± 1.4	69.9 ± 5.3* ^,^ ^†^	18.97	0.00	5.19	0.04	4.41	0.05

Note

Mean ± SE.

Abbreviations: Awak, awakenings/h; CDD, code substitution delayed memory; CDS, code substitution learning; DeSHr, desaturation events/h; Eff, sleep efficiency index; GNG, go‐no‐go; M2S, match to sample; MTH, mathematical processing; PRT, procedural reaction time; S‐HR, mean heart rate during sleep; SPD, simultaneous spatial processing; SRT, simple reaction time; SRT2, simple reaction time 2; S‐SpO2, mean SpO2 during sleep; ST6, Sternberg 6‐letter memory.

* *p* < 0.05 from SL;

† *p* < 0.05 between groups.

**TABLE A2:** Condition = Sea level versus high altitude. Group = AMS− versus AMS+. Throughput or percent correct scores of ten Automated Neuropsychological Assessment Metrics (ANAM) modules and subjective ratings of eight mood states (ANAM‐MS)

Group	AMS− (*n* = 9)		AMS+ (*n* = 10)		Condition effects		Group effects		Condition × Group	
Condition	SL	HA20	SL	HA20	*F*	*p*	*F*	*p*	*F*	*p*
*ANAM modules*										
CDS	55 ± 4	50 ± 5	59 ± 3	54 ± 5	4.12	0.06	0.57	0.46	0.09	0.76
CDD	51 ± 5	53 ± 6	51 ± 5	48 ± 5	0.01	0.94	0.18	0.67	0.44	0.52
M2S	43 ± 6	37 ± 6	29 ± 6	30 ± 6	0.57	0.46	0.57	0.46	1.84	0.19
MTH	21 ± 3	23 ± 3	24 ± 3	26 ± 3	1.82	0.19	0.60	0.45	0.17	0.68
GNG	89 ± 2	84 ± 3	89 ± 2	86 ± 3	3.73	0.07	0.07	0.80	0.41	0.53
SRT	201 ± 11	203 ± 20	220 ± 10	214 ± 19	0.02	0.88	0.66	0.43	0.11	0.75
PRT	99 ± 5	91 ± 8	96 ± 5	94 ± 8	2.07	0.17	0.00	0.99	0.70	0.41
SPD	37 ± 3	39 ± 5	31 ± 2	35 ± 4	2.38	0.14	1.10	0.31	0.18	0.68
ST6	63 ± 5	62 ± 9	65 ± 4	59 ± 8	0.56	0.46	0.00	0.95	0.15	0.70
SRT2	195 ± 13	172 ± 22	201 ± 13	188 ± 21	1.04	0.32	0.19	0.67	0.23	0.63
*Mood scale*										
Anger	0.03 ± 0.04	0.02 ± 0.26	0.14 ± 0.04	0.65 ± 0.25^†^	0.07	0.80	5.73	0.02	1.24	0.28
Anxiety	0.10 ± 0.06	0.09 ± 0.18	0.08 ± 0.06	0.42 ± 0.17	1.51	0.24	2.14	0.16	1.93	0.18
Depression	0.00 ± 0.03	0.00 ± 0.24	0.10 ± 0.03^†^	0.63 ± 0.23^†^	1.48	0.24	9.34	0.01	0.56	0.46
Fatigue	0.39 ± 0.15	0.58 ± 0.39	0.60 ± 0.14	2.17 ± 0.37^*†^	.42	0.52	6.52	0.02	5.31	0.03
Happiness	2.45 ± 0.57	1.86 ± 0.53	1.41 ± 0.54	0.72 ± 0.50	3.31	0.08	1.59	0.22	0.06	0.80
Restlessness	0.08 ± 0.05	0.22 ± 0.28	0.08 ± 0.05	0.78 ± 0.27*	6.87	0.02	1.67	0.21	2.10	0.16
Vigor	1.77 ± 0.46	1.35 ± 0.27	1.74 ± 0.44	0.45 ± 0.26*	7.34	0.01	0.83	0.37	1.57	0.23
Sleepiness	2.4 ± 0.4	2.7 ± 0.5	2.4 ± 0.4	4.8 ± 0.4* ^,^ ^†^	10.43	0.01	6.34	0.02	7.54	0.01

Note

Mean ± SE.

Abbreviations: CDD, code substitution delayed memory; CDS, code substitution learning; GNG, go‐no‐go; M2S, match to sample; MTH, mathematical processing; PRT, procedural reaction time; SPD, simultaneous spatial processing; SRT, simple reaction time; SRT2, Simple reaction time 2; ST6, Sternberg 6‐letter memory.

* *p* < 0.05 from SL;

† *p* < 0.05 between groups.

**TABLE A3:** Condition = Sea level versus high altitude. group = Sleep− versus Sleep+. Throughput or percent correct scores of 10 Automated Neuropsychological Assessment Metrics (ANAM) modules and subjective ratings of eight mood states (ANAM‐MS)

Group	Sleep+ (*n* = 7)		Sleep− (*n* = 12)		Condition effects		Group effects		Condition × Group	
Condition	SL	HA20	SL	HA20	*F*	*p*	*F*	*p*	*F*	*p*
*ANAM modules*										
CDS	56 ± 4	53 ± 5	58 ± 3	51 ± 4	3.20	0.09	0.00	0.98	0.46	0.51
CDD	55 ± 5	60 ± 6	48 ± 4	45 ± 4	0.02	0.90	3.39	0.08	0.83	0.37
M2S	47 ± 7	46 ± 6	28 ± 5	26 ± 5	0.67	0.42	6.82	0.02	0.77	0.39
MTH	26 ± 3	25 ± 4	23 ± 3	20 ± 3	1.26	0.27	0.93	0.35	0.92	0.35
GNG	91 ± 2	90 ± 4	88 ± 2	83 ± 3	2.57	0.12	3.02	0.10	0.94	0.35
SRT	224 ± 12	229 ± 22	203 ± 9	197 ± 17	0.00	0.96	1.91	0.19	0.20	0.66
PRT	104 ± 6	104 ± 9	94 ± 4	87 ± 7	1.18	0.29	2.45	0.14	1.09	0.31
SPD	37 ± 3	43 ± 5	32 ± 2	34 ± 4	3.15	0.09	2.61	0.13	0.80	0.38
ST6	69 ± 5	78 ± 9	61 ± 4	50 ± 7^†^	0.07	0.80	5.31	0.03	5.02	0.04
SRT2	210 ± 15	221 ± 22	186 ± 11	157 ± 17^†^	0.42	0.52	5.76	0.03	1.82	0.19
*Mood scale*										
Anger	0.04 ± 0.05	0.56 ± 0.31	0.12 ± 0.04	0.35 ± 0.23	0.02	0.88	7.01	0.02	3.13	0.10
Anxiety	0.13 ± 0.07	0.22 ± 0.21	0.07 ± 0.05	0.29 ± 0.16	1.63	0.22	0.00	0.97	0.10	0.75
Depression	0.04 ± 0.04	0.17 ± 0.30	0.06 ± 0.03	0.43 ± 0.23	1.34	0.26	1.64	0.22	0.02	0.89
Fatigue	0.42 ± 0.18	0.93 ± 0.53	0.55 ± 0.13	1.70 ± 0.40	0.26	0.62	0.88	0.36	0.31	0.58
Happiness	1.92 ± 0.67	1.84 ± 0.62	1.89 ± 0.51	0.92 ± 0.47	2.49	0.13	01.49	0.24	0.58	0.48
Restlessness	0.11 ± 0.06	0.17 ± 0.32	0.06 ± 0.04	0.72 ± 0.24*	4.11	0.06	0.78	0.39	3.43	0.08
Vigor	1.83 ± 0.53	1.43 ± 0.31	1.71 ± 0.40	0.56 ± 0.24* ^,^ ^†^	5.69	0.03	4.12	0.05	0.99	0.33
Sleepiness	2.36 ± 0.4	2.57 ± 0.6	2.42 ± 0.3	4.50 ± 0.4* ^,^ ^†^	6.24	0.02	5.67	0.03	5.04	0.04

Note

Mean ± SE.

Abbreviations: CDD, code substitution delayed memory; CDS, code substitution learning; GNG, go‐no‐go; M2S, match to sample; MTH, mathematical processing; PRT, procedural reaction time; SPD, simultaneous spatial processing; SRT, simple reaction time; SRT2, Simple reaction time 2; ST6, Sternberg 6‐letter memory.

* *p* < 0.05 from SL;

† *p* < 0.05 between groups.
